# The *RSPO2* gene is associated with bilateral anterior amelia in Chihuahuas

**DOI:** 10.1007/s00335-025-10123-1

**Published:** 2025-03-25

**Authors:** Lucie Chevallier, Marin Green, Julia Vo, Karen Vernau, Denis J. Marcellin-Little, Vidhya Jagannathan, Tosso Leeb, Danika Bannasch

**Affiliations:** 1 INSERM, UPEC, Ecole Nationale Vétérinaire d’Alfort, U955 – IMRB, Team 10 - Biology of the Neuromuscular System, Maisons-Alfort, France; 2 Department of Population Health and Reproduction, School of Veterinary Medicine, University of California-Davis, Davis, CA USA; 3 Department of Surgical and Radiological Sciences, School of Veterinary Medicine, University of California-Davis, Davis, CA USA; 4 Institute of Genetics, Vetsuisse Faculty, University of Bern, 3001 Bern, Switzerland

## Abstract

Bilateral anterior amelia (BAA) is the congenital absence of thoracic limbs and has been reported in the Chihuahua as an autosomal recessive disorder. In some cases, the digits of the pelvic limbs can be variably affected, but otherwise, the pelvic limbs are generally spared. A GWAS performed with nine BAA affected Chihuahuas identified a significant association on chromosome 13, and homozygosity mapping delineated a 2.1 Mb chromosomal region containing the *RSPO2* gene. Loss of function variants of *RSPO2* in humans and cattle has been associated with the absence of all limbs. Six affected Chihuahuas were whole genome sequenced (WGS) and aligned to the CanFam4 assembly. SNVs, small indels, and structural variants within the critical interval that fitted a recessive model were investigated. Three SNVs (NC_049234.1:g.8891861C > T; NC_049234.1:g.8974204C > T and NC_049234.1:g.9789424G > A) were homozygous in five cases and absent from 3,418 genetically diverse control genome sequences, except for one Small Poodle that was heterozygous. One SNV resided in RSPO2’s second intron, while the two others were intergenic. The three candidate variants were genotyped in 7 additional cases and 100 control Chihuahuas. Twelve of 13 cases were homozygous for the mutant allele, and one case was heterozygous. Controls were either homozygous for the reference allele (97%) or heterozygous (3%). Our data should facilitate genetic testing of Chihuahuas to prevent the unintentional production of BAA affected dogs. Moreover, the identification of these variants enhances understanding of *RSPO2* gene function in limb development.

**Supplementary Information:**

The online version contains supplementary material available at 10.1007/s00335-025-10123-1.

## Introduction

Congenital upper limb malformations are relatively common in humans, with an incidence of about 1 in 600 newborns (Oberg et al. 2010). These malformations vary widely, from minor alterations in digit anatomy to severe reduction defects, such as the partial or complete absence of bones (Goldfarb et al. 2020; Zuniga et al. 2012). The causes of these anomalies are diverse, including genetic mutations, chromosomal abnormalities, teratogens, vascular disruptions, or remain unknown in approximately one third of cases (McGuirk et al. 2001; Sanders and Stephens 1991; Wilkie 2003).

Limb deficiencies can be categorized as longitudinal, where part of the bone is absent, or transverse, where the limb resembles an amputation stump due to the absence of all elements beyond a certain level. Transverse deficiencies vary in severity and are classified based on which bones are missing. They develop as a failure of formation of the limb bud at a certain stage, which determines the amplitude of the anomaly (Frantz and OʼRahilly 1961; Oberg et al. 2010; Rayan and Upton 2014; Zuniga et al. 2012).

Bilateral anterior amelia (BAA), which belongs to transverse deficiencies, is characterized by the complete absence of bones distal to the scapulae (Rayan and Upton 2014). Different studies evaluated the prevalence of all types of amelia (upper and lower, unilateral and bilateral) to  ~ 1.5 per 100000 births (including live births, stillbirths, and elective terminations of pregnancy for fetal anomalies). One-third of the reported amelia cases were isolated cases, while two-thirds were associated with other congenital anomalies. Among the nonsyndromic cases, bilateral upper amelia accounted for 20% of the cases. Bilateral anterior amelia is usually associated with other malformations, and affected infants have very poor survival prognoses (Bermejo-Sánchez et al. 2011; Froster-Iskenius and Baird 1990).

Limb development in mammals is highly conserved and regulated by the coordinated expression of numerous genes whose spatio-temporal expression must be finely tuned (McQueen and Towers 2020; Tickle 2015). Disruptions in these genetic pathways can result in significant abnormalities, such as BAA (Manouvrier-Hanu et al. 2012; Oberg et al. 2010; Zuniga et al. 2012). Over 150 genes have been associated with congenital limb malformation in humans (da Rocha et al. 2021; Elsner et al. 2021; Jourdain et al. 2020; Manouvrier-Hanu et al. 2012; Sun et al. 2021; Zuniga et al. 2012), but only a few genes have been shown to be involved in severe limb reduction such as amelia. To date, *TBX5* has been associated with upper amelia, *TBX4* with lower amelia, and *WNT3*, *WNT7A* and *RSPO2* with tetra-amelia syndromes in humans (and cattle, for *RSPO2*) (Becker et al. 2020; Eyaid et al. 2011; Niemann et al. 2004; Kariminejad et al. 2009; Ranganath et al. 2020). In mouse and zebrafish models, *Fgf8*, *Fgf10*, *Fgf24*, *Sp8* as well as *HoxA* and *HoxD* gene clusters have been associated with various forms of amelia (Bell et al. 2003; Fischer et al. 2003; Kmita et al. 2005; Lewandoski et al. 2000; Min et al. 1998).

BAA in dogs is a rare phenotype. It was first documented in the 1960s in France in pointers, where experimental breeding demonstrated an autosomal recessive mode of inheritance (Ladrat et al. 1969). Later, in the 1980s, cases of bilateral anterior amelia and hemimelia were reported in Chihuahuas in Mexico. Studies on two affected families showed high phenotypic variability. Affected offspring born from healthy parents and inbreeding loops suggested an autosomal recessive transmission in that breed (Alonso et al. 1982).

This study aimed to identify the genetic determinants of BAA in Chihuahuas through a genome-wide association study (GWAS) combined with whole-genome sequencing. Identifying variants associated with BAA will enable carrier detection, aid in selective breeding to prevent affected offspring, and contribute to a deeper understanding of limb development.

## Methods

### Sample collection

DNA was extracted from 1 to 2 mL of EDTA blood using the Qiagen Puregene Blood Kit according to the manufacturer’s instructions. The DNA concentration and purity were determined by spectrophotometry (NanoDrop ND1000, Thermo Scientific), and samples were stored at  ≤ − 20 °C. Additional dog DNA samples from the Bannasch lab DNA repository at UC Davis were also included in the study (see Online Resource 1 for a detailed listing of the dogs). Familial relationships among the dogs were investigated. Information about the phenotype status of the parents and littermates was reviewed when available. No formal pedigrees were available because all dogs were adopted from shelters and rescue organizations. Due to their morphological appearance, we describe the dogs as Chihuahuas. However, they were not registered as purebred Chihuahuas with the American Kennel Club (AKC).

### Phenotyping

Thirteen BAA affected dogs were recruited for this study. The phenotype was based on the owner’s report, photos, and videos for 9 of the cases. Three cases underwent clinical examination at the UC Davis Veterinary Medical Teaching Hospital, and two of them underwent thoracic and pelvic X-rays. One case underwent clinical examination and thoracic X-rays at Desert Veterinary Clinic, Richland, WA, USA. Control Chihuahuas and Chihuahua mixes were healthy or treated for other diseases, which had no indication in their medical record of limb abnormalities.

### Genome-wide association study (GWAS)

Thirteen cases and 61 control dogs (see Online Resource 1 for a detailed listing of the dogs) were genotyped with the CanineHD BeadChip array (Illumina Inc) containing 220853 markers. Control dogs were Chihuahuas or Chihuahua mixes or other small mixes. The null hypothesis tested in this GWAS was that there is no difference in allele frequency between affected and control dogs. Since affected cases were not randomly sampled from the general Chihuahua population but instead selected based on disease status, the results should be interpreted as identifying genetic differences between this specific subset of affected dogs and the control group, rather than estimating population-wide risk allele frequencies. The CanFam3.1 assembly was used as a reference. Quality control (QC) on genotyping data was performed using the program PLINK (v1.07), (Purcell et al. 2007). Samples with missingness  > 10% were removed, and only SNVs with a minor allele frequency greater than 5% and a genotyping rate greater than 90% were included. The final dataset included a total of 74 dogs and 186,147 SNV markers. Among the 13 cases, seven dogs were related (see Online Resource 1 for family relatedness among the cases). Only one dog from each family was kept for analysis. GWAS was performed on nine unrelated cases and 61 controls using a univariate mixed model with a standardized relatedness matrix in GEMMA v.0.97 (Zhou and Stephens 2012), and likelihood ratio test *p*-values (P-lrt) were used to determine significantly associated markers. A Bonferroni-corrected significance threshold (α = 0.05) was calculated using all SNVs included in the analysis. Genomic inflation factor, as a measure of controlling for population genetic structure, was calculated in R. Manhattan and QQ plots were generated in R Statistical Software (v. 4.3.2) using qqman and dplyr libraries (R Core Team 2021).

### Short-read whole genome sequencing

Six cases and 131 control dogs from various breeds (see Online Resource 1 for a detailed list of dogs) were whole genome sequenced. Library preparation and 2 × 150 bp Illumina paired-end sequencing were performed at the UC Davis DNA Technologies Core. The raw read data was filtered using HTStream (version 1.3.3) (Petersen et al. 2015) which included screening for contaminants (such as PhiX), overlapping reads, quality-based trimming, and adapter trimming. BWA MEM (version 0.7.17) (Li and Durbin 2010) and Samtools (v. 1.14) (Li et al. 2009) were used to align the processed data to the dog reference genome assembly CanFam4 (UU_Cfam_GSD_1.0). Sequencing duplicates were marked using Picard tools (version 2.26.11) (broadinstitute.github.io/picard/). Variant calling was accomplished using the GATK (v. 4.2.5.0) (McKenna et al. 2010) pipeline which included the following steps: 1. HaplotypeCaller to call variants in the gVCF format, 2. GenomicsDB to create a datastore to store the variant call data, 3. GenotypeGVCFs to perform joint genotyping on all samples. SnpEff (v. 5.1) (Cingolani et al. 2012) was used to add effect prediction to the final VCFs using GSD_1.0 annotation (Wang et al. 2021). Alignments of the reads covering the first intron of the *RSPO2* gene on the dog reference genomes’ CanFam5 and CanFam6 (Halo et al. 2021; Vidhya Jagannathan et al. 2021) and on a grey wolf reference genome mCanLor1.2 (Sinding et al. 2021) were performed using the Geneious software (v. 2022.2.2).

### Long-read whole genome sequencing

High molecular weight DNA was isolated from a fresh EDTA blood sample of the BAA-affected dog case #2 using the HMW Nanobind kit (Pacific Biosciences, Menlo Park, CA, USA). A HIFI SMRTbell library was constructed and run on one SMRT cell 25 M on a PacBio Revio instrument. A total of 97.6 gigabases (40.69X) were generated from 6.4 million HiFi reads. The reads had a mean length of 15.2 kb and a median length of 15.3 kb, with an N50 of 18 kb. The sequencing quality was high, with a median of Q30.7 and a mean of Q24.3. Reads with a length of  < 500 kb and an average Phred score of less than 15 were filtered with Nanoflt (De Coster et al. 2018). The filtered reads were then used for assembly with Hifiasm (-t 32 -k 61 -D 4.5 –purge-max 55 -s 0.65 –primary) (Cheng et al. 2021).

### Variant filtering

Filtering of candidate variants was performed using PLINK (v. 1.07) (Purcell et al. 2007). All SNVs and small indels within the critical interval that fitted a recessive model were investigated. Variants having a mutant allele frequency of 1 in the 5 cases that showed a homozygous haplotype were filtered on 3,418 control genomes (excluding Chihuahuas), which were either internal controls produced during other projects of our group (see Online Resource 1 for a detailed listing of the dogs) or that were publicly available (Dog10K, Jagannathan et al. and Plassais et al.) (V. Jagannathan et al. 2019; Meadows et al. 2023; Ostrander et al. 2019; Plassais et al. 2019). Only variants showing a frequency < 0.01 and not homozygous for the mutant allele in any control were retained as candidates.

### Structural variant inspection

The critical interval was also analyzed for structural variants by visual inspection of the alignment files of BAA cases compared to controls using Integrative Genomics Viewer (IGV) (Robinson et al. 2011).

### In silico analysis of variants

The variant impact was analyzed in silico using the Ensembl Variant Effect Predictor tool (McLaren et al. 2016). Nucleotide conservation across different vertebrate species was evaluated using Multiz Alignment and conservation scores were evaluated using 100 vertebrates Basewise Conservation by PhyloP on the UCSC Genome Browser (Nassar et al. 2023). Prediction of transcription factor binding sites in Human genome assembly Hg38 was evaluated using JASPAR Core 2024 through UCSC Genome Browser (Rauluseviciute et al. 2024). Transcription start site, promoter, and enhancer locations were evaluated using the Dog Genome Annotation (DoGA) track hub on the UCSC Genome Browser (Hörtenhuber et al. 2024).

### Genotyping

Sanger sequencing was used to confirm the presence and to genotype the three candidate variants ((1) NC_049234.1:g.8891861C > T; (2) NC_049234.1:g.8974204C > T and (3) NC_049234.1:g.9789424G > A) in the cases (n = 13) as well as control Chihuahuas (n = 100). Toy and Miniature Poodles were also genotyped (n = 43). All primers were designed using Primer3 software (Untergasser et al. 2012)). Variants (1) and (3) were amplified by PCR from genomic DNA using primers detailed in Online Resource 2 and HotStar Taq Polymerase kit (Qiagen, Hilden, Germany) with a protocol consisting of maintenance at 95 °C for 5 min followed by 35 cycles of 94 °C for 30 s, 58 °C or 60 °C respectively for 30 s and 72 °C for 30 s, and finally maintained at 72 °C for 10 min. Variant (2) was amplified by PCR from genomic DNA using primers detailed in Online Resource 2 and Q5 High-Fidelity DNA Polymerase kit (New England Biolabs, Ipswich, MA) with a protocol consisting of maintenance at 98 °C for 30 s followed by 35 cycles of 98 °C for 10 s, 64 °C for 30 s and 72 °C for 10 s, and finally maintained at 72 °C for 2 min. After treatment with shrimp alkaline phosphatase and endonuclease I, PCR products were sent for Sanger sequencing (Genewiz, Azenta Life Sciences). Chromatogram files (ab1 files) were visualized using Chromas 2.6.6 software (Technelysium Pty Ltd, South Brisbane, QLD, Australia).

### Dog breed composition identification

Buccal swabs soaked in DNA solution (750 ng of DNA in 200 μl of elution buffer) were submitted to Embark Breed identification DNA test (Embark Veterinary Inc., Ithaca, NY, USA). The panel includes genotyping for  > 200000 genetic markers variants that indicate a dog’s breed composition.

## Results

### Phenotype description

**Fig. 1:**
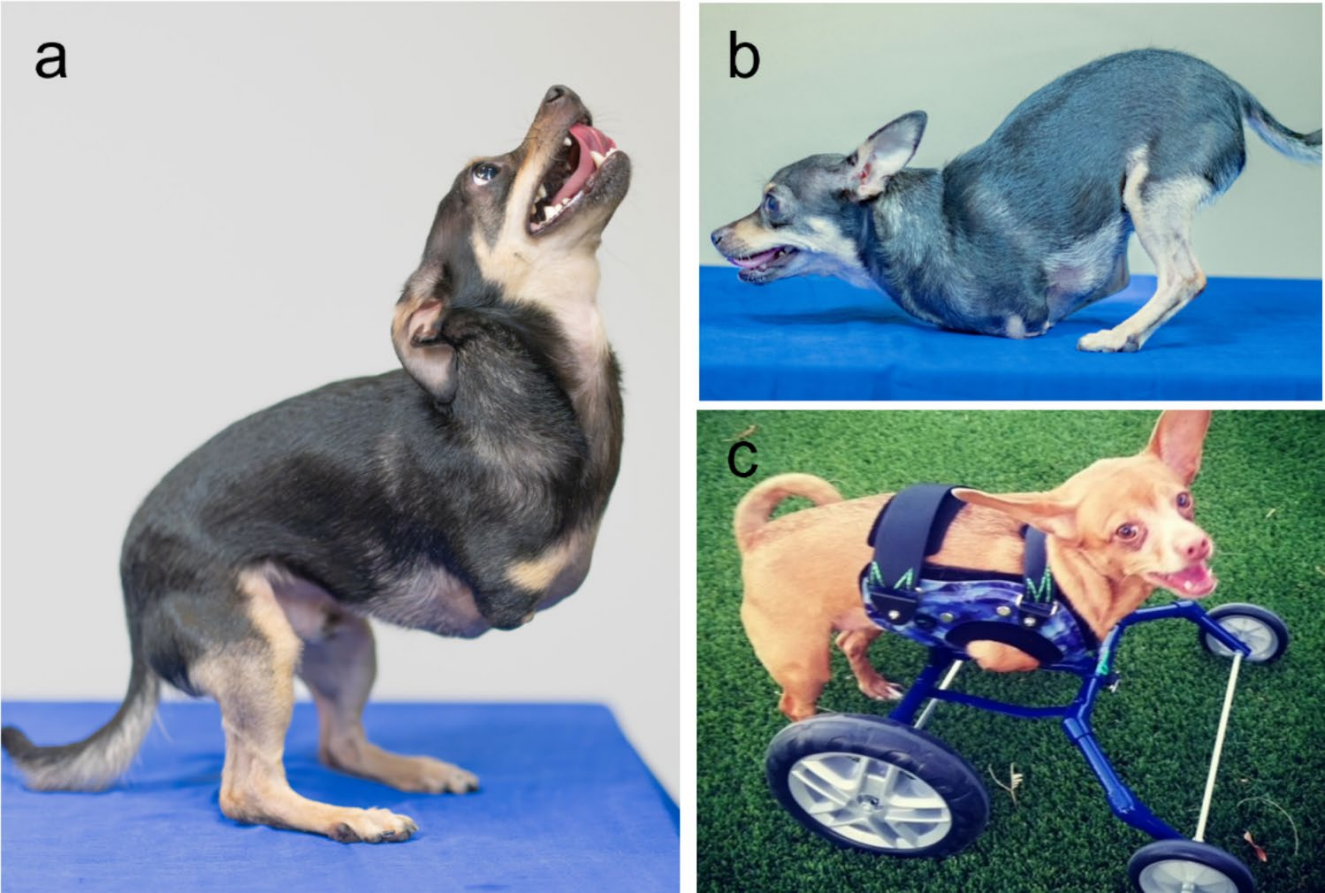
Chihuahuas affected by bilateral anterior amelia (BAA) Forelimbs were completely absent while hindlimbs were generally spared. **a** and **b** depict the same dog (case #2). Affected dogs tended to be plantigrade when the dogs stood on their hindlimbs. They moved mainly on the thorax, pushing off with their hind legs. **c** Some dogs were fitted in an ambulation cart, facilitating their ambulation (case #13)

**Fig. 2:**
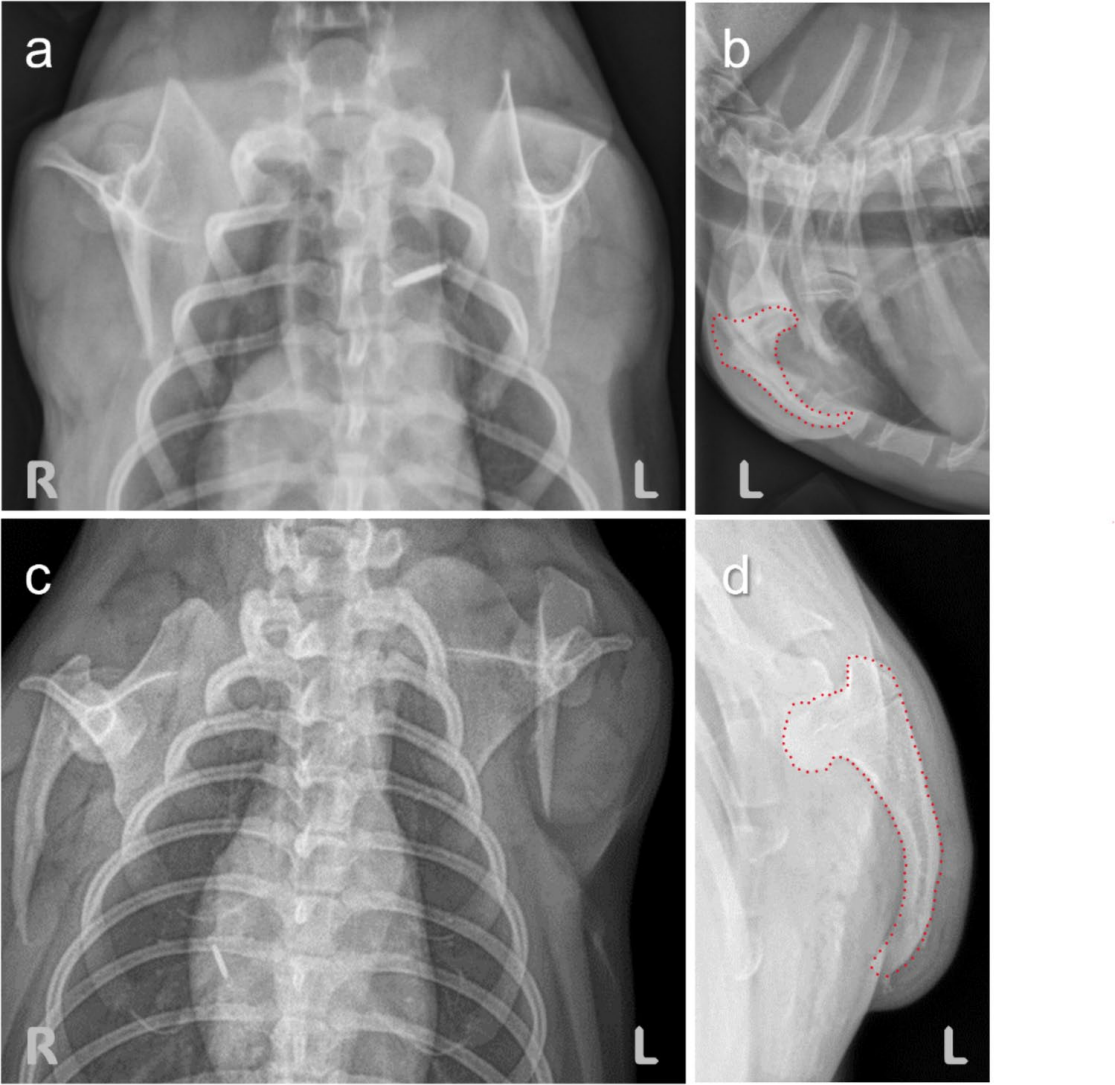
Thoracic radiographs of BAA affected Chihuahuas Scapulae are present and normally developed. Humeri are shortened and are of various lengths between individuals. Bones distal to the humeri are missing. L: left, R: right. **a** Sternal view of thorax. Humeri measuring 45 mm in length on the left side and 28 mm on the right side are seen on this dog (case #2). **b** Side view of thorax (case #2)”. The shortened humerus is underlined in red. **c** Shortened humeri measuring ~ 50 mm with a pointed tip in another dog (case #4). **d** Enlarged view of the left humerus (outlined in red) (case #4)

All the dogs in the cohort (cases #1 to #13) were Chihuahua-like (n = 6) or mixed-breed Chihuahuas (n = 7) born with no forelimbs (Fig. 1). They all had a scapula on both sides and presented a residual fragment of the humerus engaged in the glenoid cavity that varied in length (Fig. 2). All of them were missing all bones distal to the humerus. The affected dogs were generally standing in sternal decubitus (Fig. 1b) or erect on their hindlimbs, relying on the whole foot (i.e., tarsus, metatarsus, and phalanges) to stand up (Fig. 1a). Some affected dogs could be placed in ambulation carts, facilitating ambulation (Fig. 1c).

Three dogs underwent clinical examination at UC Davis Veterinary Medical Teaching Hospital (cases #1, #2, and #3), two of which underwent thoracic and pelvic X-rays (cases #1 and #2), and one dog was examined at Desert Veterinary Clinic (Richland, WA) and underwent thoracic radiographs (case #4).

One affected crossbreed Chihuahua/Dachshund female dog underwent clinical examination at UC Davis Veterinary Medical Teaching Hospital (case #1). The dog had very short palpable humeri with no motion detected in the shoulder joint. Forelimbs were entirely subcutaneous and non-functional. The dog had normal scapulae, but both humeri were markedly shortened, with only the humeral head appreciated bilaterally. The musculoskeletal structures distal to the humeral heads were bilaterally absent. On thoracic radiographs, the humeri measured approximately 10 mm in length. The dog also had bilateral mild coxofemoral joint incongruity and osteoarthrosis, as well as bilateral medial patellar luxation. For both hindlimbs, the third and fourth toes were hypotrophic, with very small nails on the left third digits and no nails on the right third and fourth digits (a picture of the hindlimbs is displayed in Online Resource 3). She was standing with pelvic limbs partially flexed and abducted. She was walking on her pelvic limbs with support from the chest. The pelvic limbs were used with a relatively wide stance and with the hip, stifle, and hock more flexed than in normal locomotion. She could stand on both pelvic limbs for a brief period. When placed in an ambulation cart, she was able to move.

Her son (case #2), resulting from an accidental backcross with her sire, was also presented for clinical examination at UC Davis Veterinary Medical Teaching Hospital. In this dog, both humeri were foreshortened with abrupt tapering and ending of the bones in the region of the proximal diaphysis. Both humeral proximal metaphyses were thin and curving caudally before their termination. The humerus measured approximately 45 mm on the right and 28 mm on the left (Fig. 2a, b). The shoulder joint could flex but did not appear to actively extend. The structures distal to the shortened humeri of both thoracic limbs were absent. Pelvic limbs were normal, with all toes present except for digit 1 (dewclaws). Toenails were present but showed abnormal growth. The dog was standing with pelvic limbs partially flexed and abducted and the chest on the ground. He could also stand solely on his pelvic limbs with the spine oblique for brief periods. He was walking on pelvic limbs with support from the chest. As in his mother, the pelvic limbs were used with a relatively wide stance, and the hip, stifle, and hock were more flexible than during normal locomotion. Besides these congenital abnormalities, this dog had recurrent aspiration pneumonia.

The third dog (case #3) presented for clinical examination at UC Davis Veterinary Medical Teaching Hospital was a male Chihuahua displaying normal subcutaneous scapulae and shortened humeri. Bones distal to humeri were absent. Pelvic limbs did not show any abnormalities. No radiographs were available for that dog. Besides these developmental abnormalities, the dog had tracheal collapse and left medial patellar luxation. The owner reported that the dog had two brothers affected by BAA and a sister with four limbs but with pigmentation disorder, generalized alopecia, craniofacial abnormalities, various additional skeletal abnormalities, and abnormal nail shape. No information was known about the phenotype of the parents.

The dog examined at Desert Veterinary Clinic (Richland, WA) (case #4) presented with the same morphological features as the three dogs described above. On thoracic radiographs, both humeral proximal metaphyses were thin and curved. The humerus measured approximately 50 mm on both sides (Fig. 2c, d). Pictures of the dog sent by the owner showed that the two middle phalanges (III and IV) of the right hindlimb looked shortened with no nails. The left hindlimb could not be assessed. The owner of the dog performed the Embark breed identification test. Breed heritage was half Chihuahua and half other breeds (Pomeranian, Border Collie, Shih Tzu, Small Poodle, Dachshund, and Pug), with both parents predicted to be mixed breed Chihuahuas.

### BAA mode of inheritance

**Fig. 3:**
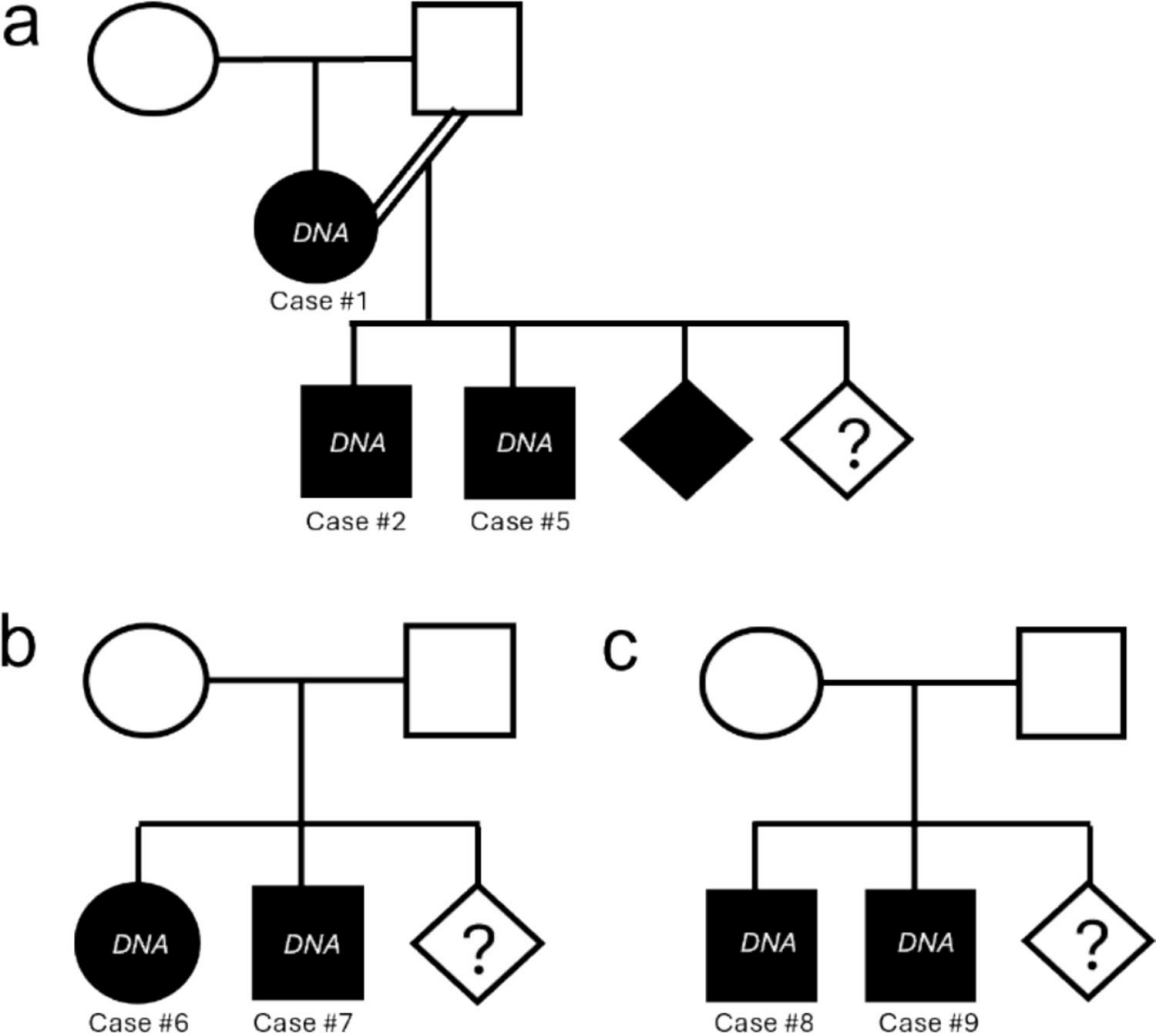
Pedigrees of the three families observed in our cohort Squares indicate males, circles indicate females, and diamonds indicate unknown sex. Filled symbols indicate affected dogs. Unfilled symbols indicate unaffected animals. The question mark indicates that the number of animals is unknown. Dogs for which DNA has been collected are identified by a white “*DNA*” symbol. **a** In this family, the dam was backcrossed with her sire, generating at least 3 affected puppies and an undetermined number of unaffected ones. **b** and **c** Two other families were characterized by unaffected parents with unknown relatedness having given birth to at least two affected puppies and an undetermined number of unaffected ones

The dogs in this cohort were adopted from rescue groups or shelters where they had been relinquished or found abandoned. Four females and nine males were sampled. None had a formal pedigree, and information on their ancestry was generally scarce or non-existent. Within this cohort, three families were identified (Fig. 3). In the family described in Fig. 3a, an affected bitch (case #1) was bred to her healthy father and produced at least three affected offspring (among which case #2 and case #5) and an undetermined number of unaffected offspring. The bitch and two of her sons were sampled for the study.

Two other pairs of siblings were also sampled. Information concerning the parents’ phenotype is unknown, but we assumed the parents had four limbs (Fig. 3b, c) since owners are unlikely to breed two-legged dogs. Male dogs with BAA would additionally not be able to breed naturally.

The appearance of BAA male and female dogs descended from healthy parents and families with inbreeding loops suggests an autosomal recessive mode of transmission.

### Mapping of the BAA locus

#### Genome wide association study

**Fig. 4:**
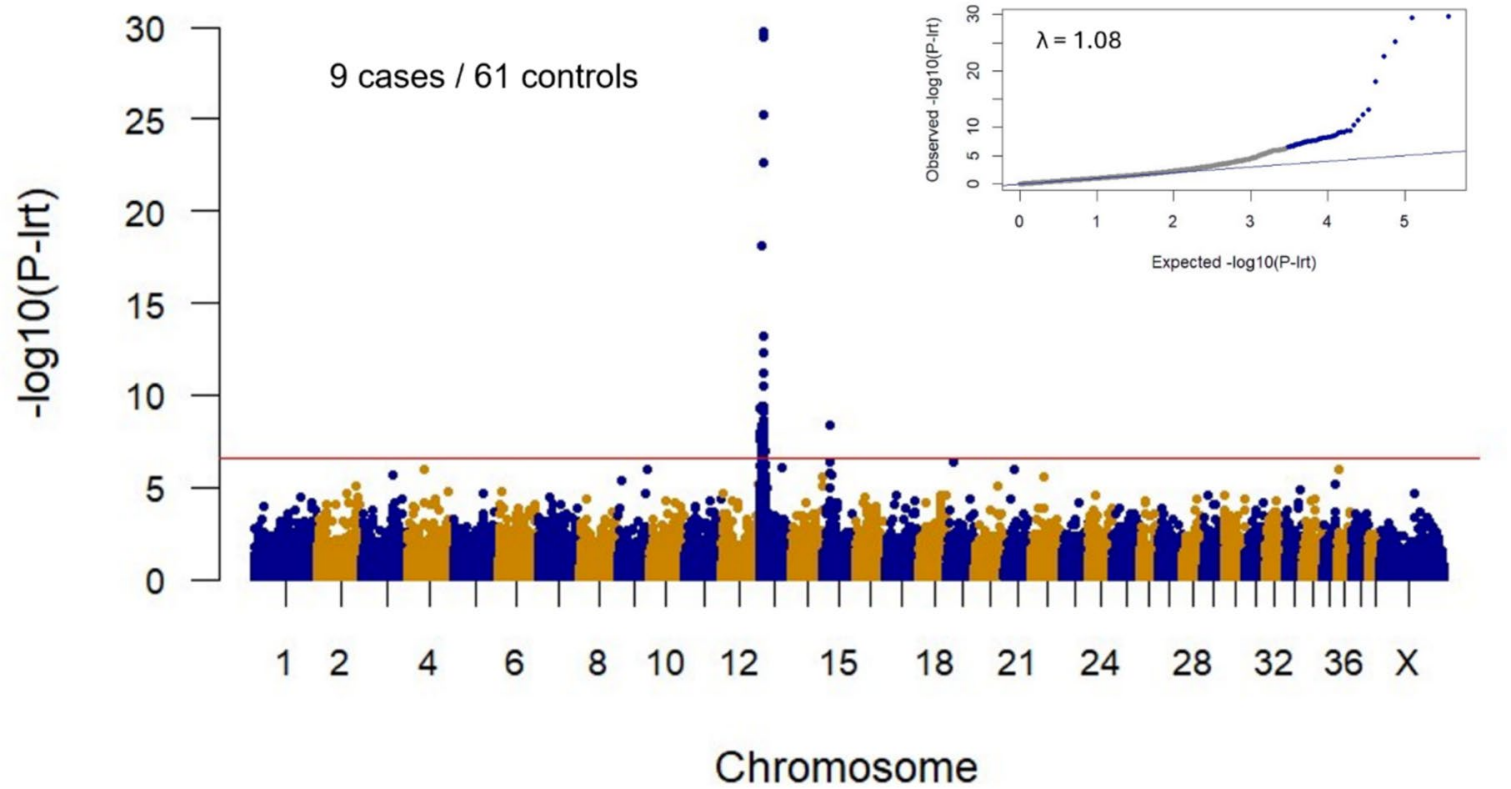
Results of the genome-wide association study A GWAS was performed in a cohort of 9 BAA cases and 61 control dogs. The red line indicates the Bonferroni-corrected genome-wide significance threshold (P_Bonf_ = 2.68 × 10^–7^). The Manhattan plot shows a strong significant signal at the beginning of chromosome 13. The quantile–quantile (QQ) plot in the inset shows the observed versus expected -log(P_lrt_) values. The straight blue line in the QQ plot indicates the distribution of P_lrt_ values under the null hypothesis. Observed P_lrt_ values above the significance threshold have been highlighted in blue. The deviation of *p*-values on the right side indicates that these markers are more strongly associated with the trait than would be expected by chance. The genomic inflation factor (λ) was 1.08

GWAS was performed using 9 cases and 61 controls and 186,147 markers. Control dogs were Chihuahuas, Chihuahua mixes, and other small mixes (see Online Resource 1 for a detailed list of dogs). A significant association was identified at the beginning of chromosome 13, with 60 markers exceeding the Bonferroni-corrected genome-wide significance threshold (P_Bonf_. = 2.68 × 10^−7^). The best associated SNV in this GWAS was on CFA13 at 9,389,470 bp (CanFam3.1 assembly), with a likelihood ratio test *p*-value of 1.89 × 10^−30^. One marker on chromosome 15 also reached the significance threshold (p-value = 4.23 × 10^–9^). A Q–Q plot of expected and observed chi-squared values indicated that population stratification was successfully controlled (λ = 1.08) (Fig. 4).

**Fig. 5:**
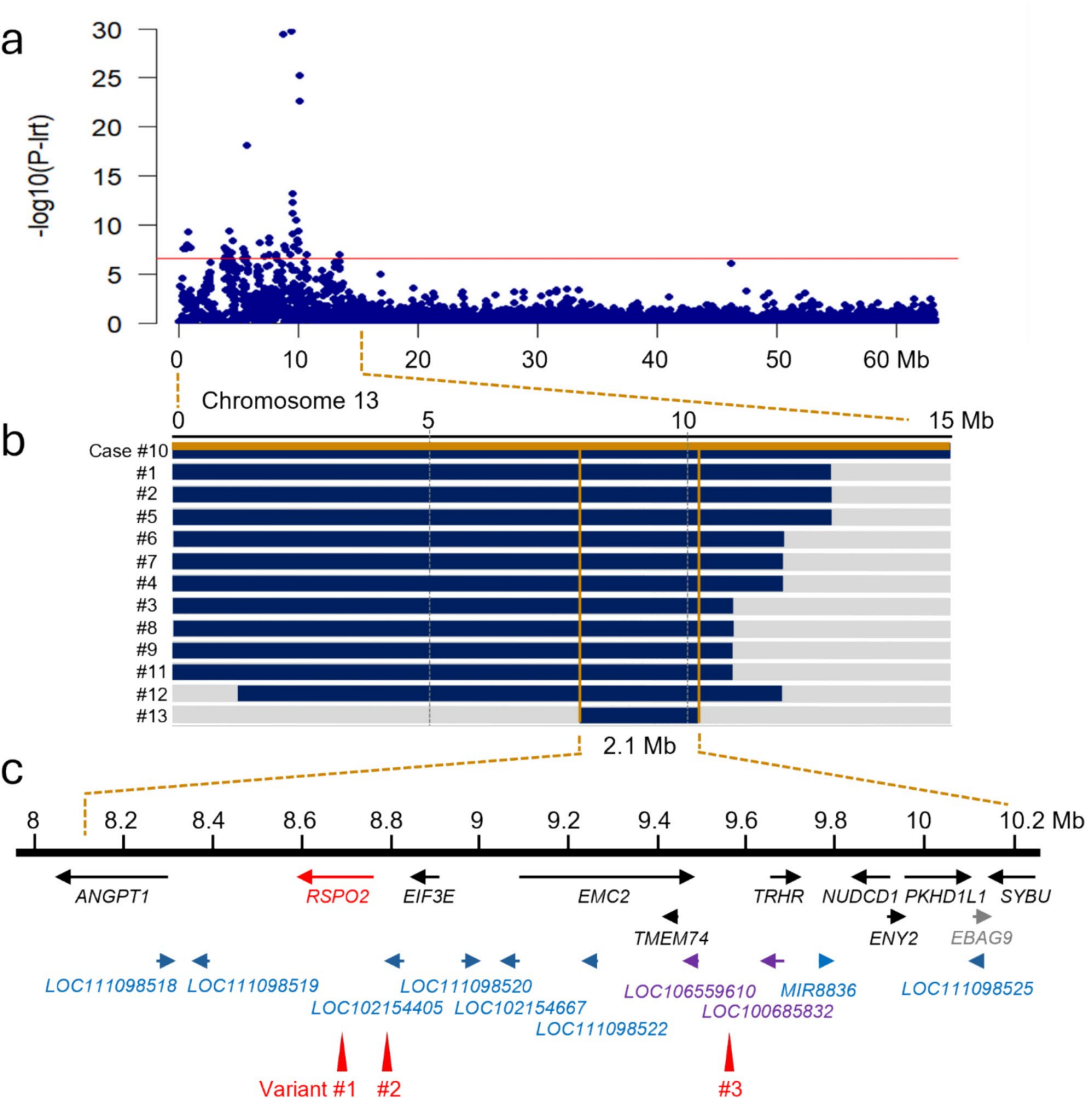
Mapping the BAA locus **a** Detailed view of GWAS results for chromosome 13. The sixty SNVs that exceed the Bonferroni-corrected genome-wide significance threshold (P_Bonf_ = 2.68 × 10^–7^) span the interval between 0.43 Mb and 13.47 Mb. **b** Haplotype analysis in the 13 BAA cases. Each horizontal bar represents the chromosome 13 haplotype of one dog. Twelve BAA affected dogs had large homozygous intervals with allele sharing on chromosome 13 (indicated in blue); one of the cases was heterozygous for the whole critical interval (case #10, indicated in blue and gold). The homozygous haplotype segment shared between the 12 homozygous dogs spanned ~ 2.1 Mb. This critical interval corresponded to the interval between the first flanking heterozygous markers on either side in a double recombinant dog, namely chr13: 8,094,036–10,172,338 (CanFam 3.1 assembly). **c** Gene annotation for the critical interval and position of three main candidate variants within the interval. The NCBI *Canis lupus familiaris* annotation release 105 listed 10 protein-coding genes (indicated in black or red), 1 provisional protein coding gene (indicated in grey), 8 genes for non-coding RNAs (indicated in blue), and 2 pseudogenes (indicated in purple). Candidate variants were numbered for simplicity: (1) Chr13:8,678,707 C > T; (2) Chr13:8,758,435 C > T and (3) Chr13:9,559,658 G > A. Bp positions are given relatively to CanFam3 to keep coherence with the whole figure. Variant (1) is located in the 2nd intron of *RSPO2*, variant (2) is intergenic between *RSPO2* and *EIF3E* and variant (3) is intergenic between *TMEM74* and *TRHR*

Significantly associated markers located on chromosome 13 spanned a ~ 13 Mb interval, from 428,328 bp to 13,470,564 bp (Fig. 5a).

#### Haplotype analysis

Homozygosity mapping was performed by visually inspecting the genotypes of cases in the region of association. A homozygous region of  ~ 2.1 Mb with allele sharing was identified in 12 cases. One case was heterozygous for this haplotype (case #10). The critical interval for BAA was defined as the interval between the first flanking heterozygous markers on either side of the shared homozygous region in the 12 cases (chr13:8,094,036–10,172,338) (Fig. 5b). The NCBI *Canis lupus familiaris* annotation release 105 listed 10 protein-coding genes and one provisional protein-coding gene, eight genes for non-coding RNAs, and two pseudogenes within this interval (Fig. 5c). Detailed list is provided in Online Resource 4.

### Identification of candidate causal variants

We sequenced the genomes of 6 BAA affected Chihuahuas (average coverage 29.5X) and called single nucleotide variants (SNVs) and small indel variants with respect to the reference genome CanFam4. Automated calling identified 40,102,010 variants in the 6 affected dogs and 131 internal control dogs from various breeds. We first filtered for variants lying within the critical interval on chromosome 13, reducing the number of candidates to 33200. Then, based on the homozygosity mapping in cases, variants were filtered that were homozygous in the 5 cases and that were either homozygous or heterozygous in case #10. This step reduced the number of candidate variants to 2,806. We then compared these variants to whole genome sequence data of 3,418 control dogs from genetically diverse breeds excluding Chihuahuas (130 internal controls, see Online Resource 1 for detailed list of dogs, and 3,288 controls dogs from publicly available datasets (Dog10K, Plassais et al. 2019 and Jagannathan et al. 2019) (V. Jagannathan et al. 2019; Meadows et al. 2023; Ostrander et al. 2019; Plassais et al. 2019). Excluding variants that were found in breeds other than Chihuahua left us with no variants.

As the BAA phenotype is rare but has already been described in other breeds, such as the pointer (Ladrat et al. 1969), we repeated the variant filtering, modifying our criteria. Starting with the 2860 variants that were found to be homozygous in the 5 cases and heterozygous or homozygous in case #10, we applied a less stringent filter, leaving open the possibility that dogs of other breeds could carry the mutant allele at a low frequency. In this way, we filtered out all variants that were homozygous in the 3,418 control dogs and accepted a heterozygote frequency of less than 0.01 among the controls. This filtering left us with 10 variants. Four of them were present in  > 20 dogs from more than 10 different breeds, as well as in village dogs and wolves with different origins, and were also found in the 4 Chihuahuas included in the public database published by Jagannathan et al. (2019). Of the 6 remaining variants, 3 were present in four dogs from four different breeds and 3 were only present in a Small Poodle. A control Chihuahua originating from the USA was also found to be heterozygous for these last 6 variants (Online Resource 5 presents a detailed list of the 10 remaining SNVs). We considered the three variants found to be homozygous in the BAA affected dogs, heterozygous in one control Chihuahua from Jagannathan et al. 2019 dataset, and heterozygous in one Small Poodle from our internal controls as the best candidate variants ((1) NC_049234.1:g.8891861C > T; (2) NC_049234.1:g.8974204C > T and (3) NC_049234.1:g.9789424G > A) (Online Resource 6 displays variants coordinates in the different dog genome assemblies). The filtering steps are summarized in Table 1. Genomic positions of these three variants relative to the genes within the BAA critical interval are depicted in Fig. 5c.

**Table 1:** Variants detected by whole genome sequencing of BAA affected dogs

Variants filtering steps	Number of variants
All variants (6 cases/131 internal controls)	40,102,010
Within critical interval	33,200
Homozygous in 5 cases	2,806
With a low allele frequency (< 0.01) AND not homozygous in 3,418 control dogs^a^	10
Only present in breeds of the same breed group (Toy)	3

^a^Control Chihuahuas were excluded from the analysis, leaving 130 internal controls, 1985 Dog10K control dogs, 584 control dogs from Jagannathan et al. 2019 and 719 control dogs from Plassais et al. 2019

**Fig. 6:**
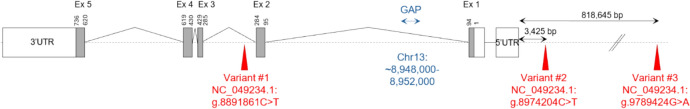
Schematic representation of the RSPO2 gene in Canis familiaris and candidate variants’ position and coverage gap position relative to *RSPO2* Positions of the three best candidate variants are depicted with red vertical arrows. Variant 1 is located in the second intron of RSPO2. Variant 2 is located about 3.4 kb upstream of the gene, while variant 3 is intergenic, about 0.82 Mb upstream of *RSPO2.* A horizontal blue double-arrow depicts the localization of a gap of ~ 4 kb in the CanFam4 assembly within the first intron of the RSPO2 gene. Please note that the orientation is with respect to the genome reference sequence and opposite to the direction of transcription. The figure is not drawn to scale

We also visually searched for structural variants within the critical interval. Inspection on IGV software of the short-read alignments of the 6 BAA affected dogs within the critical interval did not reveal any structural variants that were not present in at least one of the 130 internal control genomes (Chihuahua excluded). Nevertheless, a 4 kb gap in the CanFam4 assembly was present within the first intron of the *RSPO2* gene (from  ~ 8,948,000 to ~ 8,952,000 bp with respect to CanFam4 assembly) (Fig. 6 and Online Resource 6 which displays variants coordinates and gap coordinates in the different dog genome assemblies). Reads from the cases and from three control dogs (one Nova Scotia Duck Tolling Retriever, one Small Poodle, and one Chihuahua) were realigned on CanFam5, CanFam6, and mCanLor1.2 assemblies. Gaps varying in size were still present in these different assemblies (this repetitive region spanned about 5 kb in CanFam5, 33 kb in CanFam6, and 140 bp in mCanLor1.2). Visual inspection of SNVs, indels, and structural variants using the IGV software did not reveal any difference between cases and controls.

We obtained PacBio HIFI data at 40.69X coverage of E731. Visual inspection of the long-read alignments to the CanFam4 reference assembly of the *RSPO2* gene did not reveal any additional structural variants with respect to the short-read sequencing data. An attempt to perform a de novo assembly of the gap region in the *RSPO2* gene with the PacBio HIFI data failed (Online Resource 7 shows the assembled contig from the Hifiasm).

### Variants localization, predicted impact and conservation

Variant 1 (NC_049234.1:g.8891861C > T) was located in intron 2 of the *RSPO2* gene. This nucleotide was not conserved across different vertebrate species. The phyloP score for this nucleotide was 0.24, near-zero scores representing positions evolving at a neutral rate. The Dog Genome Annotation (DoGA) Project identified a transcription start site 672 bp away from variant 1. Variant 2 (NC_049234.1:g.8974204C > T) was intergenic, upstream of *RSPO2*, at a distance of 2,742 to 3,425 bp depending on the *RSPO2* isoform that was considered. This nucleotide was lying within a LINE-1 element. It was not conserved across different vertebrate species either and PhyloP score for this nucleotide was -1.55, negative scores indicating accelerated evolution. Variant 3 (NC_049234.1:g.9789424G > A) was intergenic, between *TMEM74* and *TRHR* genes. This SNV was located within a SINE and, therefore, not conserved (Table 2).

**Table 2:** Conservation at the genomic positions of the three candidate variants, presence of repetitive or mobile elements and predicted transcription start sites, promoters or enhancers

		Variant 1 NC_049234.1:g.8891861C > T	Variant 2 NC_049234.1:g.8974204C > T	Variant 3 NC_049234.1:g.9789424G > A
PhyloP conservation score	Score	− 0.24	− 1.55	ND
Nucleotide conservation across species	**Dog**	**C**	**C**	**G**
	Human	T	T	ND
	Rhesus	T	ND	ND
	Mouse	T	T	ND
	Elephant	A	_	ND
	Horse	**C**	ND	ND
	Rat	ND	**C**	ND
Repetitive or mobile elements	Dog CanFam4 assembly	None	LINE-1	SINE
Predicted TSSs, promoters, enhancers from DoGA	Dog CanFam4 assembly	TSS 672 bp away (8,891,095–8,891,189)	None	None

ND = Not Determined, the variant position cannot be found in human Hg38 assembly or the mouse mm10 assembly; TSS(s) = Transcription Start Site(s)

All variants (1), (2), and (3) were considered to have a potential “MODIFIER” impact by the Ensembl Variant Effect Predictor tool. The position of the three variants relative to the *RSPO2* gene is depicted in Fig. 6.

### Genotype–phenotype association

We confirmed the presence of the mutant allele at the 3 candidate variants in a homozygous state by PCR and Sanger sequencing in the 6 dogs affected by BAA that were whole genome sequenced. We also genotyped the 7 other cases. Of the 13 cases, 12 were homozygous for the mutant allele for the three candidate variants, and 1 was heterozygous (case #10). This result was expected since case #10 had two different haplotypes along the entire critical interval. This dog’s breed heritage was assessed using Embark breed identification DNA test. The test revealed that he was a mixed breed dog, half Chihuahua and half a mix of other toy breeds (Pomeranian, Shih-Tzu, American Eskimo dog, Pekingese, and other undetermined breeds), with Chihuahua ancestors on both maternal and paternal lineages. We also genotyped 100 control Chihuahuas (Online Resource 1 for a detailed listing of the dogs) for the three variants. No control Chihuahua was homozygous for the mutant allele. Three dogs were heterozygous at variants (1) and (2); all others were homozygous for the reference allele. Two dogs were heterozygous at variant (3), while the 98 others were homozygous for the reference allele. Since the three variants were found in a heterozygous state in a Small Poodle, we additionally genotyped 42 Miniature and Toy Poodles. All of them were homozygous for the reference alleles at the three variant positions (Table 3). A breed identification DNA test confirmed that the Small Poodle found to be heterozygous for the three candidate variants was a purebred Small Poodle (Miniature or Toy).

**Table 3:** Genotype frequencies in BAA cases and controls

Breed	Phenotype	No (%) Mut/Mut	No (%) + /Mut	No (%) + / +	Total
Chihuahua	Cases	12 (92.3)	1 (7.6)	0 (0)	13
	Controls (variants 1 and 2)	0 (0)	3 (3)	97 (97)	100
	Controls (variant 3)	0 (0)	2 (2)	98 (98)	100
Poodle	Controls	0 (0)	1 (2.3)	42 (97.7)	43

## Discussion

This study identified the *RSPO2* locus as a compelling candidate for bilateral anterior amelia (BAA) in Chihuahua-like dogs. The phenotype in affected dogs is quite unusual, characterized by forelimb absence or truncation and slight malformations of phalanges and nails in pelvic limbs, without additional malformations (Alonso et al. 1982). This contrasts with syndromic forms of limb agenesis observed in other species, where limb defects often accompany systemic anomalies (Aftimos and Winship 1999; Elsner et al. 2021; Eyaid et al. 2011; Kariminejad et al. 2009; Michaud et al. 1995; Niemann et al. 2004; Pelluard-Nehmé et al. 2007; Pierri et al. 2000; Powers et al. 2010; Price et al. 1998; Ranganath et al. 2020; da Rocha et al. 2021; Rosenak et al. 1991; Szenker-Ravi et al. 2018; Zuniga et al. 2012).

The disease locus was unambiguously mapped using a genome-wide association study and homozygosity analysis to a 2.1 Mb critical interval on chromosome 13. Whole genome sequencing identified three single nucleotide variants associated with the phenotype. Homozygosity for the variants was observed in 12 out of 13 affected dogs, suggesting an autosomal recessive mode of inheritance, though a semi-dominant mode of inheritance cannot be excluded. No structural variants were detected within the interval, but a gap present in all available canine genome assemblies—located in the first intron of *RSPO2*—may harbor the causative variant.

Among the genes present within the critical interval, *RSPO2* emerged as the best candidate due to its well-documented role in limb formation across vertebrates (Aoki et al. 2008; Becker et al. 2020; Bell et al. 2008; Nam et al. 2006, 2007a, b; Nam et al. 2007a, b; Szenker-Ravi et al. 2018; Tatsumi et al. 2014; Yamada et al. 2009). *RSPO2* encodes R-spondin 2, a secreted ligand that enhances the Wnt signaling pathway, by preventing the degradation of Wnt receptors, leading to β-catenin stabilization and activation of the limb developmental program (Kazanskaya et al. 2004; Nam et al. 2007a, b; Nam et al. 2007a, b; Srivastava et al. 2024; ter Steege and Bakker 2021). The Wnt/β-catenin pathway is integral to maintaining the apical ectodermal ridge (AER), a key signaling center that is at the apex of the limb bud and is responsible for proximo-distal limb outgrowth (McQueen and Towers 2020; Tickle 2015). In the mouse, *Rspo2* is strongly expressed in the AER and in the mesenchymal cells proximal to the trunk during limb development, and the study of *Rspo2*-null mice showed that *Rspo2* was required for AER maintenance (Aoki et al. 2008; Kazanskaya et al. 2004; Nam et al. 2007a, b; Yamada et al. 2009). Abrogation of AER leads to transverse defects that correspond to the time of disruption (Oberg et al. 2010).

Studies on *Rspo2*-null or hypomorphic alleles in various species provide compelling evidence for *Rspo2*′s pivotal role in limb development (Aoki et al. 2008; Becker et al. 2020; Bell et al. 2008; Nam et al. 2007a, b; Szenker-Ravi et al. 2018; Tatsumi et al. 2014; Yamada et al. 2009). In cattle and humans, large deletions or nonsense variants in *RSPO2* cause tetra-amelia, characterized by the complete absence of all four limbs and lung aplasia (Becker et al. 2020; Szenker-Ravi et al. 2018). Studies of recessive *RSPO2*-null variants in humans have demonstrated reduced Wnt potentiation proportional to allele severity. The causal link between gradual *RSPO2* deficiency and absence of limb segments was furthermore demonstrated by injecting unilaterally *Rspo2* guide RNA with Cas9 protein into *Xenopus tropicalis* embryos at the two-cell stage, which caused marked unilateral forelimb and hindlimb amelia (Szenker-Ravi et al. 2018). In *Rspo2*-null mice, loss of β-catenin signaling impairs AER maintenance, leading to severe limb deformities, such as truncated hindlimbs or absence of some bones, absent digits, and absence of ossification in the forelimbs. Notably, some alleles exhibit asymmetrical limb deformities, indicating potential regional specificity in *Rspo2* regulation. *Rspo2*-null mice die shortly after birth, exhibiting other skeletal anomalies, such as craniofacial deformities, and suffer from lung and kidney development anomalies (Aoki et al. 2008; Bell et al. 2008; Nam et al. 2007a, b; Yamada et al. 2009). Similarly, *Rspo2*-null zebrafish display severe morphological defects, including the absence of dorsal and anal fins (Tatsumi et al. 2014). These findings collectively underscore *RSPO2*’s critical and conserved role in both forelimb and hindlimb development.

Additionally, a 167 bp insertion in the 3′ UTR of the *RSPO2* gene has been shown to cause the furnishing phenotype in dogs (Cadieu et al. 2009). Even though this variant does not affect limb development, it demonstrates that non-coding variants in *RSPO2* can lead to visible morphological traits. The variants identified in this study may disrupt *RSPO2* expression by altering transcriptional regulation. One hypothesis is that these variants interfere with binding sites or create aberrant sites for transcription factors crucial to *RSPO2* expression in forelimb buds, leading to localized gene downregulation and early AER disruption (Nam et al. 2007a, b; Oberg et al. 2010; VanderMeer and Ahituv 2011). Alternatively, the causative variant may reside in the uncovered genomic gap within *RSPO2*′s first intron. That region, characterized by repetitive elements and structural complexity, could harbor regulatory elements essential for forelimb-specific gene expression.

Unlike RSPO2 deficient models, which exhibit multisystem abnormalities and early lethality, BAA-affected dogs display isolated forelimb defects. This suggests that these variants generate a hypomorphic allele that specifically disrupts *RSPO2* expression during early forelimb development while sparing other developmental processes. The dog model thus provides a rare opportunity to consider isolated forelimb amelia and offers an interesting resource for investigating *RSPO2*’s region-specific effects on limb development.

The cases examined in this study exhibited a complete absence of forelimbs, a trait readily identifiable by rescue groups, shelters, and owners. However, it is likely—if not certain—that the phenotypic spectrum is broader, as described in the original publication of the phenotype (Alonso et al. 1982) and also supported by observations of the sister of case #3, for example. Milder manifestations, such as missing digits or even just nails, may go undetected. Thus, this cohort supports an autosomal recessive mode of inheritance for the condition, but further study of genotype–phenotype correlation involving more comprehensively phenotyped Chihuahua and Chihuahua-related dogs could refine this understanding. Notably, the presence of a heterozygous affected dog raises the possibility that the disease may exhibit a semi-dominant inheritance pattern. In some cases, heterozygosity for the identified variants, combined with other variants affecting R-spondin receptors or Wnt pathway interactors, may disrupt the fine-tuning of Wnt/β-catenin signaling, resulting in amelia or related phenotypic variations (Srivastava et al. 2024; ter Steege and Bakker 2021). Investigating the BAA phenotype variability may deepen our understanding of *RSPO2* alleles and their modifiers’ effect on limb development.

This study may also have practical implications for breeding management in the Chihuahua breed. Although the estimated carrier frequency is relatively low (approximately 3%), implementing genetic testing could help prevent the unintentional birth of affected dogs.

## Supplementary Information

Below is the link to the electronic supplementary material.

Supplementary file1 (XLSX 27 KB)

Supplementary file2 (XLSX 10 KB)

Supplementary file3 (TIF 1960 KB)

Supplementary file4 (XLSX 16 KB)

Supplementary file5 (XLSX 19 KB)

Supplementary file6 (XLSX 19 KB)

Supplementary file7 (PNG 275 KB)

## Data Availability

Whole-genome sequencing files reported in this paper can be found in the NCBI Sequence Read Archive (SRA Bioproject no. PRJNA961733; PRJNA377155; PRJNA776905), see Online Resource 1 for detailed information.

## References

[CR1] Aftimos S, Winship I (1999) A patient with VACTERL association, amelia and hemifacial microsomia. Clin Dysmorphol 8(2):135–137 10319203

[CR2] Alonso RA, Hernández A, Díaz P, Cantú JM (1982) An autosomal recessive form of hemimelia in dogs. Vet Rec 110(6):128–129. 10.1136/vr.110.6.128 7186700 10.1136/vr.110.6.128

[CR3] Aoki M, Kiyonari H, Nakamura H, Okamoto H (2008) R-Spondin2 expression in the apical ectodermal ridge is essential for outgrowth and patterning in mouse limb development. Dev Growth Differ 50(2):85–95. 10.1111/j.1440-169X.2007.00978.x 18067586 10.1111/j.1440-169X.2007.00978.x

[CR4] Becker D, Weikard R, Schulze C, Wohlsein P, Kühn C (2020) A 50-Kb deletion disrupting the RSPO2 gene is associated with tetradysmelia in Holstein Friesian cattle. Genet Select Evolut: GSE 52:68. 10.1186/s12711-020-00586-y 10.1186/s12711-020-00586-y PMC7661195 33176673

[CR5] Bell SM, Schreiner CM, Waclaw RR, Campbell K, Potter SS, Scott WJ (2003) Sp8 is crucial for limb outgrowth and neuropore closure. Proc Nat Acad Sci 100(21):12195–12200. 10.1073/pnas.2134310100 14526104 10.1073/pnas.2134310100 PMC218735

[CR6] Bell SM, Schreiner CM, Wert SE, Mucenski ML, Scott WJ, Whitsett JA (2008) R-Spondin 2 is required for normal Laryngeal-Tracheal, lung and limb Morphogenesis. Development 135(6):1049–1058. 10.1242/dev.013359 18256198 10.1242/dev.013359

[CR7] Bermejo-Sánchez E, Cuevas L, Amar E, Bakker MK, Bianca S, Bianchi F, Canfield MA et al (2011) Amelia: a multi-center descriptive epidemiologic study in a large dataset from the international clearinghouse for birth defects surveillance and research, and overview of the literature. Am J Med Genet C Semin Med Genet 157(4):288–304. 10.1002/ajmg.c.30319 10.1002/ajmg.c.30319 PMC4453759 22002956

[CR8] Cadieu E, Neff MW, Quignon P, Walsh K, Chase K, Parker HG, Vonholdt BM et al (2009) Coat variation in the domestic dog is governed by variants in three genes. Science 326(5949):150–153. 10.1126/science.1177808 19713490 10.1126/science.1177808 PMC2897713

[CR9] Cheng H, Concepcion GT, Feng X, Zhang H, Li H (2021) Haplotype-Resolved de Novo assembly using phased assembly graphs with Hifiasm. Nat Methods 18(2):170–175. 10.1038/s41592-020-01056-5 33526886 10.1038/s41592-020-01056-5 PMC7961889

[CR10] Cingolani P, Platts A, Wang LL, Coon M, Nguyen T, Wang L, Land SJ, Xiangyi Lu, Ruden DM (2012) A program for annotating and predicting the effects of single nucleotide polymorphisms, SnpEff: SNPs in the genome of Drosophila Melanogaster strain W1118; Iso-2; Iso-3. Fly 6(2):80. 10.4161/fly.19695 22728672 10.4161/fly.19695 PMC3679285

[CR11] Coster De, Wouter SD, Schultz DT, Cruts M, Van Broeckhoven C (2018) NanoPack: visualizing and processing long-read sequencing data. Bioinformatics 34(15):2666–2669. 10.1093/bioinformatics/bty149 29547981 10.1093/bioinformatics/bty149 PMC6061794

[CR12] da Rocha L, Alves LV, Pires L, Yamamoto GL, Ceroni JRM, Honjo RS, de Novaes E, Bisneto F, Oliveira LAN et al (2021) Congenital limb deficiency: genetic investigation of 44 individuals presenting mainly longitudinal defects in isolated or syndromic forms. Clin Genet 100(5):615–623. 10.1111/cge.14041 34341987 10.1111/cge.14041

[CR13] Elsner J, Mensah MA, Holtgrewe M, Hertzberg J, Bigoni S, Busche A, Coutelier M et al (2021) Genome sequencing in families with congenital limb malformations. Hum Genet 140(8):1229–1239. 10.1007/s00439-021-02295-y 34159400 10.1007/s00439-021-02295-y PMC8263393

[CR14] Eyaid W, Al-Qattan MM, Abdulkareem IA, Fetaini N, Balwi MA (2011) A novel Homozygous Missense mutation (c.610G>A, p.Gly204Ser) in the WNT7A gene causes Tetra-Amelia in two Saudi families. Am J Med Genet A 155A(3):599–604. 10.1002/ajmg.a.33717 21344627 10.1002/ajmg.a.33717

[CR15] Fischer S, Draper BW, Neumann CJ (2003) The Zebrafish Fgf24 mutant identifies an additional level of Fgf signaling involved in vertebrate forelimb initiation. Development 130(15):3515–3524. 10.1242/dev.00537 12810598 10.1242/dev.00537

[CR16] Frantz CH, O’Rahilly R (1961) Congenital skeletal limb deficiencies. JBJS 43(8):1202–1224. 10.2106/00004623-196143080-00012

[CR17] Froster-Iskenius UG, Baird PA (1990) Amelia: incidence and associated defects in a large population. Teratology 41(1):23–31. 10.1002/tera.1420410104 2305372 10.1002/tera.1420410104

[CR18] Goldfarb CA, Ezaki M, Wall LB, Lam WL, Oberg KC (2020) The Oberg-Manske-Tonkin (OMT) classification of congenital upper extremities: update for 2020. J Hand Surg 45(6):542–547. 10.1016/j.jhsa.2020.01.002 10.1016/j.jhsa.2020.01.002 32093994

[CR19] Halo JV, Pendleton AL, Shen F, Doucet AJ, Derrien T, Hitte C, Kirby LE et al (2021) Long-read assembly of a great dane genome highlights the contribution of GC-rich sequence and mobile elements to canine genomes. Proc Natl Acad Sci USA 118(11):e2016274118. 10.1073/pnas.2016274118 33836575 10.1073/pnas.2016274118 PMC7980453

[CR20] Hörtenhuber M, Hytönen MK, Mukarram AK, Arumilli M, Araujo CL, Quintero I, Syrjä P et al (2024) The DoGA Consortium expression atlas of promoters and genes in 100 canine tissues. Nat Commun 15(1):9082. 10.1038/s41467-024-52798-1 39433728 10.1038/s41467-024-52798-1 PMC11494170

[CR21] Jagannathan V, Drögemüller C, Leeb T, Dog Biomedical Variant Database Consortium (DBVDC), Aguirre G, André C, Bannasch D, Becker D, Davis B, Ekenstedt K, Faller K (2019) A comprehensive biomedical variant catalogue based on whole genome sequences of 582 dogs and eight wolves. Anim Genet 50(6):695–704. 10.1111/age.12834 31486122 10.1111/age.12834 PMC6842318

[CR22] Jagannathan V, Hitte C, Kidd JM, Masterson P, Murphy TD, Emery S, Davis B et al (2021) Dog10K_Boxer_Tasha_1.0: a long-read assembly of the dog reference genome. Genes 12(6):847. 10.3390/genes12060847 34070911 10.3390/genes12060847 PMC8228171

[CR23] Jourdain A-S, Petit F, Odou M-F, Balduyck M, Brunelle P, Dufour W, Boussion S et al (2020) Multiplex targeted high-throughput sequencing in a series of 352 patients with congenital limb malformations. Hum Mutat 41(1):222–239. 10.1002/humu.23912 31502745 10.1002/humu.23912

[CR24] Kariminejad A, Goodarzi P, Asghari-Roodsari A, Kariminejad MH (2009) Amelia, Cleft Lip, and Holoprosencephaly: a distinct entity. Am J Med Genet A 149A(12):2828–2831. 10.1002/ajmg.a.32933 19938097 10.1002/ajmg.a.32933

[CR25] Kazanskaya O, Glinka A, del Barco I, Barrantes PS, Niehrs C, Wei Wu (2004) R-Spondin2 Is a secreted activator of Wnt/β-catenin signaling and is required for Xenopus Myogenesis. Dev Cell 7(4):525–534. 10.1016/j.devcel.2004.07.019 15469841 10.1016/j.devcel.2004.07.019

[CR26] Kmita M, Tarchini B, Zàkàny J, Logan M, Tabin CJ, Duboule D (2005) Early developmental arrest of mammalian limbs lacking HoxA/HoxD gene function. Nature 435(7045):1113–1116. 10.1038/nature03648 15973411 10.1038/nature03648

[CR27] Ladrat J, Blin P-C, Lauvergne J-J (1969) Ectromélie Bithoracique Héréditaire Chez Le Chien. Ann Genet Sel Anim 1(2):119. 10.1186/1297-9686-1-2-119

[CR28] Lewandoski M, Sun X, Martin GR (2000) Fgf8 signalling from the AER is essential for normal limb development. Nat Genet 26(4):460–463. 10.1038/82609 11101846 10.1038/82609

[CR29] Li H, Durbin R (2010) Fast and accurate long-read alignment with burrows-wheeler transform. Bioinformatics (Oxford, England) 26(5):589–595. 10.1093/bioinformatics/btp698 20080505 10.1093/bioinformatics/btp698 PMC2828108

[CR30] Li H, Handsaker B, Wysoker A, Fennell T, Ruan J, Homer N, Marth G, Abecasis G, Durbin R (2009) The sequence alignment/map format and SAMtools. Bioinformatics 25(16):2078–2079. 10.1093/bioinformatics/btp352 19505943 10.1093/bioinformatics/btp352 PMC2723002

[CR31] Manouvrier-Hanu S, Florence P, Holder-Espinasse M, Escande-Narducci F (2012) Limb development anomalies: genetics. eLS. 10.1002/9780470015902.a0005986.pub2

[CR32] McGuirk CK, Westgate M-N, Holmes LB (2001) Limb deficiencies in newborn infants. Pediatrics 108(4):e64. 10.1542/peds.108.4.e64 11581472 10.1542/peds.108.4.e64

[CR33] McKenna A, Hanna M, Banks E, Sivachenko A, Cibulskis K, Kernytsky A, Garimella K et al (2010) The genome analysis toolkit: a mapreduce framework for analyzing next-generation DNA sequencing data. Genom Res 20(9):1297–1303. 10.1101/gr.107524.110 10.1101/gr.107524.110 PMC2928508 20644199

[CR34] McLaren W, Gil L, Hunt SE, Riat HS, Ritchie GRS, Thormann A, Flicek P, Cunningham F (2016) The Ensembl variant effect predictor. Genome Biol 17(1):122. 10.1186/s13059-016-0974-4 27268795 10.1186/s13059-016-0974-4 PMC4893825

[CR35] McQueen C, Towers M (2020) Establishing the pattern of the vertebrate limb. Development 147(17):dev177956. 10.1242/dev.177956 32917670 10.1242/dev.177956

[CR36] Meadows JRS, Kidd JM, Wang G-D, Parker HG, Schall PZ, Bianchi M, Christmas MJ et al (2023) Genome sequencing of 2000 canids by the Dog10K consortium advances the understanding of demography, genome function and architecture. Genom Biol 24(1):187. 10.1186/s13059-023-03023-7 10.1186/s13059-023-03023-7 PMC10426128 37582787

[CR37] Michaud J, Filiatrault D, Dallaire L, Lambert M (1995) New autosomal recessive form of amelia. Am J Med Genet 56(2):164–167. 10.1002/ajmg.1320560210 7625439 10.1002/ajmg.1320560210

[CR38] Min H, Danilenko DM, Scully SA, Bolon B, Ring BD, Tarpley JE, DeRose M, Simonet WS (1998) Fgf-10 is required for both limb and lung development and exhibits striking functional similarity to drosophila branchless. Genes Dev 12(20):3156–3161. 10.1101/gad.12.20.3156 9784490 10.1101/gad.12.20.3156 PMC317210

[CR39] Nam J-S, Turcotte TJ, Smith PF, Choi S, Yoon JK (2006) Mouse Cristin/R-Spondin family proteins are novel ligands for the Frizzled 8 and LRP6 receptors and activate beta-catenin-dependent gene expression. J Biol Chem 281(19):13247–13257. 10.1074/jbc.M508324200 16543246 10.1074/jbc.M508324200

[CR40] Nam J-S, Park E, Turcotte TJ, Palencia S, Zhan X, Lee J, Yun K, Funk WD, Yoon JK (2007a) Mouse R-Spondin2 Is required for apical ectodermal ridge maintenance in the hindlimb. Dev Biol 311(1):124–135. 10.1016/j.ydbio.2007.08.023 17904116 10.1016/j.ydbio.2007.08.023 PMC2692258

[CR41] Nam J-S, Turcotte TJ, Yoon JK (2007b) Dynamic expression of *R-Spondin* family genes in mouse development. Gene Expr Patterns 7(3):306–312. 10.1016/j.modgep.2006.08.006 17035101 10.1016/j.modgep.2006.08.006

[CR42] Nassar LR, Barber GP, Benet-Pagès A, Casper J, Clawson H, Diekhans M, Fischer C et al (2023) The UCSC genome browser database: 2023 update. Nucleic Acids Res 51(D1):D1188–D1195. 10.1093/nar/gkac1072 36420891 10.1093/nar/gkac1072 PMC9825520

[CR43] Niemann S, Zhao C, Pascu F, Stahl U, Aulepp U, Niswander L, Weber JL, Müller U (2004) Homozygous WNT3 mutation causes tetra-amelia in a large consanguineous family. Am J Hum Genet 74(3):558–563. 10.1086/382196 14872406 10.1086/382196 PMC1182269

[CR44] Oberg KC, Feenstra JM, Manske PR, Tonkin MA (2010) Developmental biology and classification of congenital anomalies of the hand and upper extremity. J Hand Surg 35(12):2066–2076. 10.1016/j.jhsa.2010.09.031 10.1016/j.jhsa.2010.09.031 21134615

[CR45] Ostrander EA, Wang G-D, Larson G, Vonholdt BM, Davis BW, Jagannathan V, Hitte C, Wayne RK, Zhang Y-P (2019) Dog10K: an international sequencing effort to advance studies of canine domestication, phenotypes and health. Natl Sci Rev 6(4):810–824. 10.1093/nsr/nwz049 31598383 10.1093/nsr/nwz049 PMC6776107

[CR46] Pelluard-Nehmé F, Baudet C, Carles D, Alberti EM, Delrue MA, Lacombe D (2007) A new case of VACTERL association with unilateral amelia of upper limb. Clin Dysmorphol 16(3):185. 10.1097/MCD.0b013e3280fa81f1 17551334 10.1097/MCD.0b013e3280fa81f1

[CR47] Petersen, Kristen R., David A. Streett, Alida T. Gerritsen, Samuel S. Hunter, and Matthew L. Settles. 2015. “Super Deduper, Fast PCR Duplicate Detection in Fastq Files.” In *Proceedings of the 6th ACM Conference on Bioinformatics, Computational Biology and Health Informatics*, Atlanta Georgia: ACM, 491–92. 10.1145/2808719.2811568.

[CR48] Pierri NB, Lecora M, Passariello A, Scala I, Andria G (2000) New case of bilateral upper limb amelia, facial clefts, and renal hypoplasia. Am J Med Genet 91(2):123–125. 10.1002/(SICI)1096-8628(20000313)91:2%3c123::AID-AJMG8%3e3.0.CO;2-N 10748410 10.1002/(sici)1096-8628(20000313)91:2<123::aid-ajmg8>3.0.co;2-n

[CR49] Plassais J, Kim J, Davis BW, Karyadi DM, Hogan AN, Harris AC, Decker B, Parker HG, Ostrander EA (2019) Whole genome sequencing of canids reveals genomic regions under selection and variants influencing morphology. Nat Commun 10(1):1489. 10.1038/s41467-019-09373-w 30940804 10.1038/s41467-019-09373-w PMC6445083

[CR50] Powers MY, Karbe GT, Gregor TP, McKelvie P, Culp WTN, Fordyce HH, Smith GK (2010) Evaluation of the relationship between orthopedic foundation for animals’ hip joint scores and PennHIP distraction index values in dogs. J Am Vet Med Assoc 237(5):532–541. 10.2460/javma.237.5.532 20807130 10.2460/javma.237.5.532

[CR51] Price SM, Berry AC, Raymond FL, Turnpenny P, Young ID (1998) Four cases of amelia of the upper limb associated with anal atresia–is this VACTERL with extreme limb involvement? Clin Dysmorphol 7(1):35–40 9546828

[CR52] Purcell S, Neale B, Todd-Brown K, Thomas L, Ferreira MAR, Bender D, Maller J et al (2007) PLINK: a tool set for whole-genome association and population-based linkage analyses. Am J Hum Genet 81(3):559–575. 10.1086/519795 17701901 10.1086/519795 PMC1950838

[CR53] R Core Team. 2021. “R: A Language and Environment for Statistical Computing.” https://www.R-project.org/.

[CR54] Ranganath P, Perala S, Nair L, Pamu PK, Shankar A, Murugan S, Dalal A (2020) A newly recognized multiple malformation syndrome with caudal regression associated with a Biallelic c.402G>a variant in TBX4. Eur J Hum Genet 28(5):669–673. 10.1038/s41431-020-0572-5 31965066 10.1038/s41431-020-0572-5 PMC7170885

[CR55] Rauluseviciute I, Riudavets-Puig R, Blanc-Mathieu R, Castro-Mondragon JA, Ferenc K, Kumar V, Lemma RB et al (2024) JASPAR 2024: 20th anniversary of the open-access database of transcription factor binding profiles. Nucl Acids Res 52(D1):D174–D182. 10.1093/nar/gkad1059 37962376 10.1093/nar/gkad1059 PMC10767809

[CR56] Rayan GM, Upton J III (2014) Amelia/Hemimelia. Congenital hand anomalies and associated syndromes. Springer, Amsterdam, pp 173–174

[CR57] Robinson JT, Thorvaldsdóttir H, Winckler W, Guttman M, Lander ES, Getz G, Mesirov JP (2011) Integrative genomics viewer. Nat Biotechnol 29(1):24–26. 10.1038/nbt.1754 21221095 10.1038/nbt.1754 PMC3346182

[CR58] Rosenak D, Ariel I, Arnon J, Diamant YZ, Ben Chetrit A, Nadjari M, Zilberman R et al (1991) Recurrent tetraamelia and pulmonary hypoplasia with multiple malformations in sibs. Am J Med Genet 38(1):25–28. 10.1002/ajmg.1320380107 2012129 10.1002/ajmg.1320380107

[CR59] Sanders DD, Stephens TD (1991) Review of drug-induced limb defects in mammals. Teratology 44(3):335–354. 10.1002/tera.1420440310 1948768 10.1002/tera.1420440310

[CR60] Sinding M-H, Gopalakrishnan S, Raundrup K, Dalén L, Threlfall J, Gilbert T (2021) The genome sequence of the grey wolf, canis lupus linnaeus 1758. Wellcome Open Res 6:310. 10.12688/wellcomeopenres.17332.1 34926833 10.12688/wellcomeopenres.17332.1 PMC8649967

[CR61] Srivastava A, Rikhari D, Srivastava S (2024) RSPO2 as Wnt signaling enabler: important roles in cancer development and therapeutic opportunities. Genes Dis 11(2):788–806. 10.1016/j.gendis.2023.01.013 37692504 10.1016/j.gendis.2023.01.013 PMC10491879

[CR62] Sun L, Huang Y, Zhao S, Zhao J, Yan Z, Guo Y, Lin M et al (2021) Deciphering the mutational signature of congenital limb malformations. Mol Ther Nucl Acids 24:961–970. 10.1016/j.omtn.2021.04.012 10.1016/j.omtn.2021.04.012 PMC8141661 34094714

[CR63] Szenker-Ravi E, Altunoglu U, Leushacke M, Bosso-Lefèvre C, Khatoo M, Tran HT, Naert T et al (2018) RSPO2 inhibition of RNF43 and ZNRF3 governs limb development independently of LGR4/5/6. Nature 557(7706):564–569. 10.1038/s41586-018-0118-y 29769720 10.1038/s41586-018-0118-y

[CR64] Tatsumi Y, Takeda M, Matsuda M, Suzuki T, Yokoi H (2014) TALEN-mediated mutagenesis in Zebrafish reveals a role for *r-Spondin 2* in fin ray and vertebral development. FEBS Lett 588(24):4543–4550. 10.1016/j.febslet.2014.10.015 25448983 10.1016/j.febslet.2014.10.015

[CR65] ter Steege EJ, Bakker ERM (2021) The role of R-Spondin proteins in cancer biology. Oncogene 40(47):6469–6478. 10.1038/s41388-021-02059-y 34663878 10.1038/s41388-021-02059-y PMC8616751

[CR66] Tickle C (2015) How the embryo makes a limb: determination, polarity and identity. J Anat 227(4):418–430. 10.1111/joa.12361 26249743 10.1111/joa.12361 PMC4580101

[CR67] Untergasser A, Cutcutache I, Koressaar T, Ye J, Faircloth BC, Remm M, Rozen SG (2012) Primer3—new capabilities and interfaces. Nucleic Acids Res 40(15):e115. 10.1093/nar/gks596 22730293 10.1093/nar/gks596 PMC3424584

[CR68] VanderMeer JE, Ahituv N (2011) Cis-regulatory mutations are a genetic cause of human limb malformations. Dev Dyn off Publ Am Assoc Anat 240(5):920–930. 10.1002/dvdy.22535 10.1002/dvdy.22535 PMC3174732 21509892

[CR69] Wang C, Wallerman O, Arendt M-L, Sundström E, Karlsson Å, Nordin J, Mäkeläinen S et al (2021) A novel canine reference genome resolves genomic architecture and uncovers transcript complexity. Commun Biol 4(1):1–11. 10.1038/s42003-021-01698-x 33568770 10.1038/s42003-021-01698-x PMC7875987

[CR70] Wilkie AOM (2003) Why study human limb malformations? J Anat 202(1):27–35. 10.1046/j.1469-7580.2003.00130.x 12587917 10.1046/j.1469-7580.2003.00130.x PMC1571054

[CR71] Yamada W, Nagao K, Horikoshi K, Fujikura A, Ikeda E, Inagaki Y, Kakitani M et al (2009) Craniofacial malformation in R-Spondin2 knockout mice. Biochem Biophys Res Commun 381(3):453–458. 10.1016/j.bbrc.2009.02.066 19233133 10.1016/j.bbrc.2009.02.066

[CR72] Zhou X, Stephens M (2012) Genome-wide efficient mixed model analysis for association studies. Nat Genet 44(7):821–824. 10.1038/ng.2310 22706312 10.1038/ng.2310 PMC3386377

[CR73] Zuniga A, Zeller R, Probst S (2012) The molecular basis of human congenital limb malformations. Wires Dev Biol 1(6):803–822. 10.1002/wdev.59 10.1002/wdev.59 23799625

