# Supplementary figures and images for: The RSPO2 gene is associated with bilateral anterior amelia in Chihuahuas
Source: Mamm Genome. 2025 Mar 25;36(3):746–60. doi: 10.1007/s00335-025-10123-1 (PMC12408703; doi:10.1007/s00335-025-10123-1)

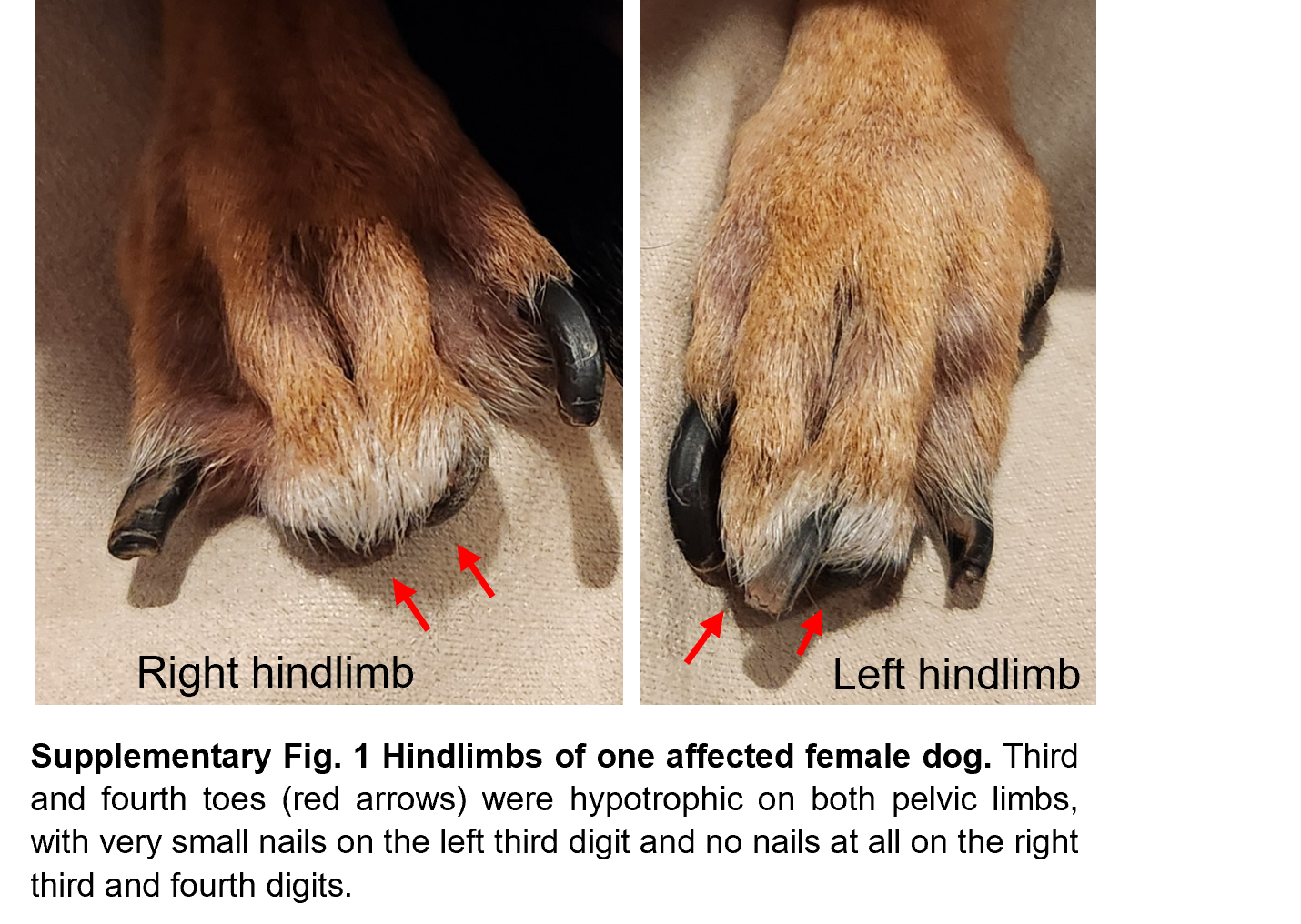

Supplement: Supplementary file 3 — Supplementary file3 (TIF 1960 KB) [file 335_2025_10123_MOESM3_ESM.tif]

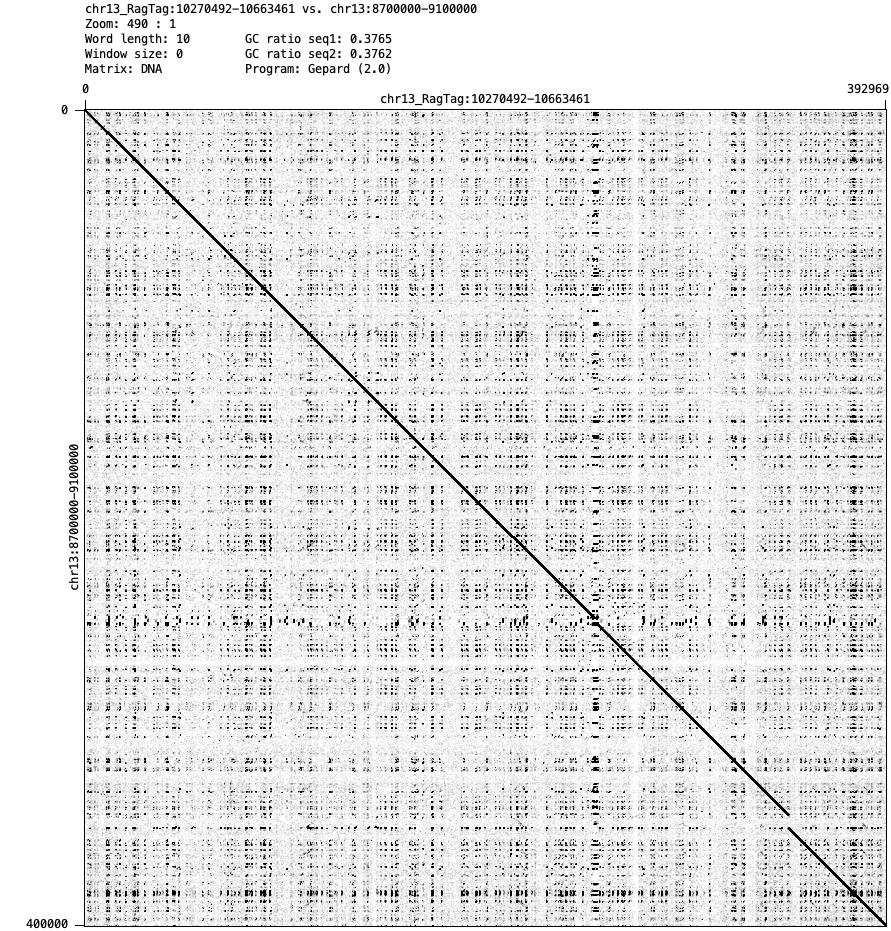

Supplement: Supplementary file 7 — Supplementary file7 (PNG 275 KB) [file 335_2025_10123_MOESM7_ESM.png]
